# Financial reimbursement - irrelevant for GPs’ readiness to implement brief intervention to reduce alcohol consumption? A cross-sectional vignette study

**DOI:** 10.1186/s12875-020-01231-9

**Published:** 2020-08-19

**Authors:** Thomas Fankhaenel, Katrin Schulz, Lars-Eric Petersen, Andreas Klement, Thomas Frese

**Affiliations:** 1 University of Halle-Wittenberg, Halle, Germany; 2 SRH University of Applied Health Sciences, Gera, Germany

**Keywords:** General practitioner, Screening and brief intervention (SBI), Excessive alcohol consumption, Financial reimbursement

## Abstract

**Background:**

General Practitioners’ (GPs) readiness to implement screening and brief intervention (SBI) to treat patients with excessive alcohol consumption is low. Several studies identified crucial barriers such as insufficient financial reimbursement. In contrast to the barriers-account, we assume that low implementation readiness of GPs may be less attributed to external barriers but rather more so to inherent characteristics of SBI. To test our assumption, we conducted a vignette study assessing the GPs’ readiness to implement SBI in comparison to a pharmacological intervention also designed for the treatment of excessive drinkers in relation to standard or above-standard financial reimbursement. According to our hypothesis GPs should be less ready to implement SBI regardless of financial reimbursement.

**Methods:**

A convenience sample of GPs was recruited to answer the questionnaire. To assess the GPs’ implementation readiness a 4-item 6-point Likert scale was developed and pretested.

**Results:**

One hundred forty GPs completed the questionnaire. GPs were more ready to implement the pharmacological intervention than SBI, *F*(1,132) = 27.58, *p* > .001 (main effect).

We found no effect for financial reimbursement, *F*(1,132) = 3.60, *ns*, and no interaction effect, *F*(1,132) = 2.20, *ns*.

**Conclusions:**

Further research should investigate more thoroughly the crucial characteristics of SBI to initiate a modification process finally leading to more effective primary care dependency prevention.

## Background

Preventive approaches such as screening and brief intervention (SBI) have been shown to be effective in reducing alcohol consumption in general practice patients [1] [2]. Brief intervention (BI) in the form of feedback, information, and advice for patients screening positive has been thus recommended to German General Practitioners (GPs) [3]. Nonetheless most GPs do not implement BI into their routine care [4] [5] [6] [7]. To identify barriers against implementation, several studies have been conducted [8] [9] [10] [11] [12] [13] [14] [15] identifying a large number of barriers subsumed to categories including organizational factors (e.g. lack of financial incentives), staff factors (e.g. lack of knowledge), patient factors (e.g. negative reaction in terms of embarrassment or unease) [1], individual level factors (lack of education), or society-level factors (heterogeneous understanding of the alcohol problem) [13]. This remarkable number of barriers can be interpreted in at least two different ways. It can be interpreted first as the disclosure of SBI as a badly designed and possibly too demanding treatment not feasible for the target group of excessive drinkers in primary care. It can also be interpreted however as an indicator of the GPs’ uncertainty regarding what prevents them from implementing a well efficacious intervention.

To advance the discourse we want to suggest a change of perspective. We believe that talking in terms of barriers implicates that an aspired activity - implementing a brief intervention for patients who screened positive - is not translated into action because of one or more barriers preventing the GP - from implementing it. If so, the elimination of those barriers should result in action and widespread implementation of SBI in primary care. However, only small effects were found by modifying external conditions such as reimbursement. Although for example an experimentally introduced reimbursement led to improved implementation (fee for service), and implementation declined after terminating the reimbursement [16], we presume nonetheless that barriers such as lack of or insufficient financial reimbursement are not the major problem. Other research seems to support this position [17] [18].

In contrast to the barriers-account we presume that inherent characteristics of BI may be understood as the primary problem. BI should be implemented in an empathetic, respectful, positive relationship with the patient. GPs should work with the person’s own ideas, concerns and motivations by using active listening skills, including open-ended questions, and affirmations in order to encourage the patients to take personal responsibility for their decisions [3]. These conditions may cause GPs to worry about being overwhelmed. For instance, GPs may be possibly forced to convince excessive drinkers to reduce their alcohol consumption by means of advice and gentle forms of persuasive communication (e.g. Motivational Interviewing [19]) in the worst case resulting in a debate with difficult to refute disagreement, hostile response, or respectively feigned compliance [15]. We assume moreover that such negative expectations may be less probable when using an intervention without those demanding characteristics. To test our hypothesis in this study half of the GPs were asked to assess their readiness to implement BI whereas the other half were asked to assess their readiness to implement a pharmacological intervention (PI) also designed to reduce the alcohol consumption of excessive drinkers (nalmefene [20]). Additionally, the effect of intervention type, BI versus PI, should be compared with the effect of an above-standard financial reimbursement. According to our hypothesis we expected that GPs would report being more ready to implement the PI regardless of financial reimbursement.

## Methods

### Design and setting

We planned a cross-sectional, randomized vignette study using a 2 (BI vs. PI) × 2 (standard reimbursement versus above standard reimbursement) design. For randomization of GPs we used a PC random generator determining the order of questionnaires. A convenience sample of GP offices in Saxony-Anhalt, Thuringia, Saxony, and Hesse was recruited by conducting a spontaneous visit by a research assistant during opening hours. In an attempt to attain our recruitment goal of 200 participants, 246 GPs practicing in four German federal states were consulted personally at their general practice offices by nine research assistants. On site, the practice nurse was informed about the study and asked about inviting their GP to participate. At the time of GPs’ consent to participate, study information, consent forms, questionnaire, and the payment-sheet were handed over to them. The completed study materials were gathered in a follow-up practice visit. Each GP completing the questionnaire received a financial reimbursement of 25 Euro.

### Questionnaire

The questionnaire was developed by an interdisciplinary team of GPs, social scientists, and psychologists experienced in their respective fields of research. It was self-designed and development followed the concept of face validity. The questionnaire was pretested by scientifically experienced GPs and the feedback led to minor modifications. A piloting was done with a subsample of eight GPs. After introduction GPs were provided with an equivalent amount of information about either BI or PI for patients with excessive alcohol consumption (first independent variable). In the BI condition GPs were informed about the procedure of screening and brief intervention including information about the AUDIT-C screener and eight randomly selected BI treatment activities such as advising the patient to drink nonalcoholic beverages if thirsty or to drink alcohol only in small sips [21]. In the PI condition GPs received information about the pharmacological intervention with nalmefene designed to help patients to reduce their alcohol consumption [22] [20]. Both questionnaires are included in the supplementary material.

GPs of both conditions then read about an identical situation describing a known patients’ visit to their general practice. They also read that the consultation raised the suspicion of an excessive alcohol consumption. Finally, GPs were asked to evaluate their readiness to implement the depicted intervention - BI versus PI- under conditions of either standard financial reimbursement or above-standard financial reimbursement (second independent variable). In the standard financial reimbursement condition one half of GPs assessed their implementation readiness in relation to a financial reimbursement of 18€ for 20 min intervention according to the German fee-for-service reimbursement standard [23]. In the above-standard financial reimbursement condition the other half of GPs were asked to assess their implementation readiness in relation to a financial reimbursement twice as high (36€) for the same length of time.

The GPs’ implementation readiness was assessed by a Likert scale ranging from 1 (do not agree) to 6 (do agree) using the following pretested items: 1) For the depicted financial reimbursement (18 vs. 36€) I would inform the patient with excessive alcohol consumption about the intervention (BI vs. pharmacological intervention); 2) For the depicted financial reimbursement (18 vs. 36€) I would carry out the intervention for the patient agreeing to participate (BI vs. pharmacological intervention); 3) The intervention (BI vs. pharmacological intervention) is not feasible in my own general practice; 4) The intervention (BI vs. pharmacological intervention) is not effective to reduce the alcohol consumption of excessive drinkers.

For control purposes the GPs’ attitude to alcohol problems and their treatment was assessed by using the Short Alcohol and Alcohol Problems Perception Questionnaire (SAAPPQ [24]). The SAAPPQ is a validated measure providing information about the GPs’ perceptions of a) the adequacy of their skills and knowledge in relation to problem drinkers (subscale role security) and b) how appropriate it is for them to engage in work with such clients (subscale therapeutic commitment) [24]. It also ranges from 1 (do not agree) to 6 (do agree).

Finally, demographic data and practice information were gathered. SPSS statistical software (version 22.0) was used to calculate means as well as standard deviations and to run AN(C) OVAs for continuous variables. A statistically significant difference was stated for *p* < 0.05.

## Results

Of the 246 GPs who were approached, 200 agreed to participate in the study. Of these, 140 GPs returned the questionnaire. All returned questionnaires were complete and could be analyzed. Mean age of GPs was 50 years (*SD* = 9.70) with a mean of 15.40 years in practice (*SD* = 9.13). More than half of the GPs were women (66.2%), 96 managed an urban practice (69.6%), and 47 worked in a group practice, in a practice sharing or were employed in an ambulatory health care center (34.1%). The GPs of our sample ran their general practices in 31 cities with a maximum of 22 GPs in Leipzig and a minimum of 1 GP in 20 cities.

GPs’ attitude to alcohol problems was assessed by the SAAPPQ questionnaire with a moderate reliability (*Cronbachs’ alpha* = .68). GPs reported a mean attitude to alcohol problems and their treatment of 4.66 (*SD* = 0.61). GPs of the BI condition did not differ markedly from GPs of the PI condition regarding age, years in practice, percentage of female participants, urban practices, and attitude towards alcohol problems. The groups differed markedly in the percentage of group practices (see Table 1), even though the group assignment had been random.

**Table 1:** GP and practice characteristics in the BI and PI condition; subgroups do not differ significantly in any characteristic

	condition	
	BI	PI
Number	61	79
Mean age in years (SD)	50 (10.25)	50 (9.33)
% women (N)	62.3 (38)	69.2 (54)
Mean years in practice (SD)	16 (9.81)	14.9 (8.61)
% urban^a^ practices (N)	72.1 (44)	67.5 (52)
% group practices^b^ (N)	43.3% (26)	26.9% (21)

^a^ Identification as ‘urban’ or ‘rural’ practice according to the GPs judgement

^b^ Practice sharing and ambulatory health care center are subsumed to group practices

Implementation readiness of GPs was assessed by a 4-item 6-point Likert scale with also moderate reliability (*Cronbachs’ alpha* = .72). GPs reported a mean implementation readiness of 4.01 (*SD* = 1.37). An ANCOVA with intervention (BI/PI) and financial reimbursement (standard/above-standard) as between-subject factors, attitude about alcohol problems (SAAPPQ) and practice type (single/group practice) as covariates and implementation readiness as dependent variable revealed a main effect for intervention, but not for financial reimbursement (see Table 2). GPs were more ready to implement the PI (*M* = 4.44, *SD* = 1.23) in comparison to the BI (*M* = 3.39, *SD* = 1.33; see Table 2). We found furthermore a positive association between the attitude to alcohol problems and the intervention readiness.

**Table 2:** ANCOVA results with main effects for intervention type (BI vs. PI) and GPs’ attitudes to alcohol problems (SAAPPQ) regarding implementation readiness

	df	F	*P*-value
Intervention (BI/PI)	1132	27.58	.000
Financial reimbursement (standard/above)	1132	3.60	.060
GPs’ attitudes to alcohol problems (SAAPPQ)	1132	6.31	.013
Practice type (single/group)	1132	3.11	.080

## Discussion

Previous research explained the GPs’ low readiness to implement BI for excessive drinkers by pointing out the large number of implementation barriers. By means of vignette study we tried to show that GPs’ low readiness should be explained rather by inherent characteristics of BI and less by associated barriers such as insufficient financial reimbursement [11]. In our vignette study we tested for the combined effect of the factors intervention type (BI vs. PI) and financial reimbursement (standard vs. above-standard) on the implementation readiness of GPs. We found that GPs’ reported readiness to implement BI was lower than their readiness to implement a pharmacological intervention. However, only a tendency that did not reach statistical significance could be found for the second factor (financial reimbursement). GPs trended toward a slightly higher implementation readiness under the condition of above-standard financial reimbursement.

To reduce alcohol consumption several approaches have been created (e.g. Motivational Interviewing [19]). These approaches have been evaluated positively by efficacy studies and are thus recommended for use [1]. It is probably also true that GPs are generally ready to motivate excessive drinkers to reduce their alcohol consumption and they probably know very well about the health costs of excessive drinking [25]. But they are nevertheless not ready to treat these patients with SBI. Although our results were only recorded in Germany, they seem to be valid internationally too. Comparative studies are available, for instance, for Switzerland and France [7], as well as Sweden and the Netherlands [26]. Both studies provide corresponding results. Although Swedish patients received more SBI than Dutch patients, only 6.0% received advice on how to reduce their alcohol consumption in comparison to 4.7% in the Netherlands. Moreover, the vast majority of 91% of French GPs did not use any test to screen for hazardous drinking in comparison to 77% of Swiss GPs. Even more daunting in this context may be the systematic review about strategies to improve the implementation of SBI [27] including results from 13 countries such as USA, Australia, GBR, and Spain showing that none of the tested strategies showed significant improvement regarding patient outcomes. We believe hence that the substantial number of barriers reported in previous studies indicates some ‘deeper’ conflict being rooted in the inherent characteristics of BI and we’d like to ask, is the time ripe now to quit forcing the implementation of BI into routine care?

It was not the intention of our study to show the superiority of pharmacological interventions to treat excessive drinkers and our results should not be understood as recommendation to prefer this type of intervention to BI. Pharmacological interventions to reduce the alcohol consumption are heavily disputed and a recommendation to use, for instance, anti-craving drugs would be inadequate considering the complexity of the issue. Nonetheless, GPs seem to perceive some advantages in using a pharmacological intervention and the question may be raised: What are those advantages and how can their understanding and a better understanding of GPs be used to improve BI? This question can hardly be answered easily. Some speculations may however be provided.

Firstly, GPs may prefer habitual behaviors [28]. Habitual behaviors are usually well-practiced, well-structured, and are mostly associated with lower risk of failure. Accordingly, GPs may be used to ending their treatment with prescribing a drug, leading to a preference for such a behavior. Time pressure and workload, quite common in general practice, may even facilitate the use of habitual behaviors. Secondly, GPs may prefer to maintain social control over the course of consultation. A drug can be prescribed in a predictable structured act without the risk of inducing a less controllable dispute about idiosyncratic barriers and costs of reducing alcohol consumption and the vague issue of a healthier lifestyle. Finally, GPs may prefer the pharmacological intervention based on their belief that such an intervention may be more effective than a mere verbal intervention like BI. They may, for instance, perceive that BI’s effectiveness depends primarily on a patient’s self-control in contrast to a pharmacological intervention influencing the organism more directly. Risky consumers are not disobedient in general and may know about the unhealthiness of their behavior. But they may have failed several times to achieve a lower consumption level leading to resignation and a low self-efficacy regarding their self-control.

There are some limitations to the present study. First, the GP’s readiness to implement BI was compared with their readiness to implement a pharmacological intervention newly available in Germany and not very common. This intervention was used in the control condition because of being also designed to reduce the alcohol consumption of excessive drinkers. The comparison of the two interventions may be perceived as problematic for some reasons. Both interventions, BI and the pharmacological intervention, may include overlapping elements such as information and advice. They may furthermore differ in several associated aspects beyond the pure mean of intervention such as duration, effectiveness, or perceived eligibility regarding the target group. GPs may hence tend to prefer one of the two also for personal reasons based on their - probably heterogeneous - past experiences with these interventions. Both interventions should not be understood thus as equivalent or comparable solutions for the problem of excessive drinking. Because of their multiple differences it’s impossible - based on our results - to attribute the GP’s lower implementation readiness to specific aspects of BI. But this was not the intention of our investigation. It was the intention to compare the effect of the intervention type - BI versus another intervention with other characteristics for the same target group - with the effect of the financial reimbursement to show the greater relevance of the factor intervention type. And this was done. A second limitation was the recruitment of GPs based on voluntary participation should be noted implicating selection bias and the risk of results with limited representativeness. Voluntary participation was implemented by the Ethics Committee of the University of Halle and is a widespread practice in this field of research. Thirdly, the following aspects may limit the generalizability of our results. As a predictor of implementation readiness, we used the self-reported intention of GPs. Self-reports, especially regarding desired or recommended behaviors such as BI, are possibly biased towards social desirability and would hence indicate a higher readiness than will be shown in routine care. However, using intention as a predictor of future behavior is an accepted practice in psychological research [29]. Its predictive validity can be increased by using items specific to the context in question. This condition was met in our study. Moreover, GPs had to assess their implementation readiness based on written information about both interventions in the artificial context of case vignettes. Written information may provide a reduced and abstracted picture of the real conditions possibly inducing a weaker effect than real world conditions would do. Case vignettes are however a valid strategy to measure clinical competence [30]. Finally, it should be mentioned that other factors may have influenced our findings, such as GPs’ concerns about the risk of stigmatization or a lack of skills. Future research should seek to clarify to what extent such factors, which have thus far been studied less, actually impair the GPs’ readiness to implement SBI.

What are the implications for practical care? In Germany, primary care dependency prevention via SBI does not work effectively because all of the efforts to increase the GPs’ use of SBI haven’t been effective so far. Past research has emphasized that barriers may prevent GPs from implementing BI into routine care. It showed also that the elimination of barriers such as insufficient financial reimbursement did not lead to widespread implementation as may be expected [16] suggesting doubts regarding the belief that even a substantial increase of financial reimbursement would produce a primary care system with systematic screening of all patients and BI if screened positive. If inherent characteristics of BI are the problem its concept should be modified. Our results suggest the idea that inherent characteristics of BI may have a more negative effect on the implementation readiness of GPs than barriers such as an insufficient financial reimbursement. We show that GPs were more ready to treat an excessive drinker with a newly and even critically disputed pharmacological intervention than to use BI. Further research should investigate the crucial characteristics of BI to initiate a modification process finally leading to more effective primary care dependency prevention.

## Conclusions

To explain the GPs’ low readiness to implement SBI into routine care some crucial barriers, as for example insufficient financial reimbursement were identified by previous research. We showed for the first time that inherent characteristics of SBI had a more negative influence on the GPs’ readiness than (insufficient) financial reimbursement. To test our assumption, we conducted a vignette study assessing the GPs’ readiness to implement SBI in comparison to a pharmacological intervention also designed for the treatment of excessive drinkers in relation to standard or above-standard financial reimbursement. We conclude thus that even improved financial reimbursement will probably not lead to the intended outcome of a generally practiced implementation of SBI in general practice. Our results suggest a modification of the SBI-concept and its inherent characteristics.

## Supplementary information

**Additional file 1.**

**Additional file 2.**

## Data Availability

The datasets used for the current study are available from the corresponding author on reasonable request.

## Abbreviations

GP: General practitioner
GPs: General practitioners
SBI: Screening and brief intervention
BI: Brief intervention
PI: Pharmacological intervention
SAAPPQ: Short alcohol and alcohol problems perception questionnaire
AUDIT-C: Alcohol use disorders identification test
ANCOVA: Analysis of covariance
