# Supplementary material for: Financial reimbursement - irrelevant for GPs’ readiness to implement brief intervention to reduce alcohol consumption? A cross-sectional vignette study
Source: BMC Fam Pract. 2020 Aug 19;21:170. doi: 10.1186/s12875-020-01231-9 (PMC7439686; doi:10.1186/s12875-020-01231-9)
Supplement: Supplementary file 1 — Additional file 1. [file 12875_2020_1231_MOESM1_ESM.pdf]

Gefördert durch:
Bundesministerium für Gesundheit
aufgrund eines Beschlusses des Deutschen Bundestages

Martin-Luther-Universität Halle-Wittenberg

FACHHOCHSCHULE FÜR GESUNDHEIT
SRH GERA

Fb-Nr - _ _ _ -

Dear Colleague,

We would like to thank you for supporting us in our research.

For years, the World Health Organization (WHO) and other institutions have called on General Practitioners to get more involved in the treatment of patients with problematic alcohol consumption. The goal of our research is a critical review of presently available treatment options for those patients with special attention to feasibility in routine care. We would like to know what you think about the presently available treatment options designed for general practice.

Filling out our questionnaire takes around 10 minutes.
Please complete our questionnaire from front to back.

We are at your disposal for any questions.

Prof. Dr. phil. Thomas Fankhänel [1,2], Prof. Dr. med. Andreas Klement [1]

[1] Martin-Luther-Universität Halle-Wittenberg
Medizinische Fakultät, Sektion Allgemeinmedizin
Magdeburger Str. 8, D-06112 Halle

[2] Studiengang Gesundheitspsychologie
SRH Fachhochschule für Gesundheit Gera
Neue Straße 28-30, 07548 Gera
Telefon: +49 365 773407-41
Telefax: +49 365 773407-77

For years, the WHO and other institutions have called on General Practitioners (GPs) to get more involved in the treatment of patients with excessive alcohol consumption.

GPs should systematically screen all of their patients for their alcohol consumption levels by means of a screening questionnaire.

Such a screening questionnaire is the AUDIT-C shown on the next page. The AUDIT-C consists of three questions, each of which asks about the alcohol consumption habits of the patient.

When a patient reaches four or more points (termed hazardous alcohol consumption) he should be treated with a brief intervention.

On the page after the next page you will find an example of such a brief intervention as it was developed by the Bundeszentral für gesundheitliche Aufklärung (BzgA).

Please take a close look at both pages.

Please turn the page!

Fig. 1: AUDIT-C Screening questionnaire for alcohol consumption.

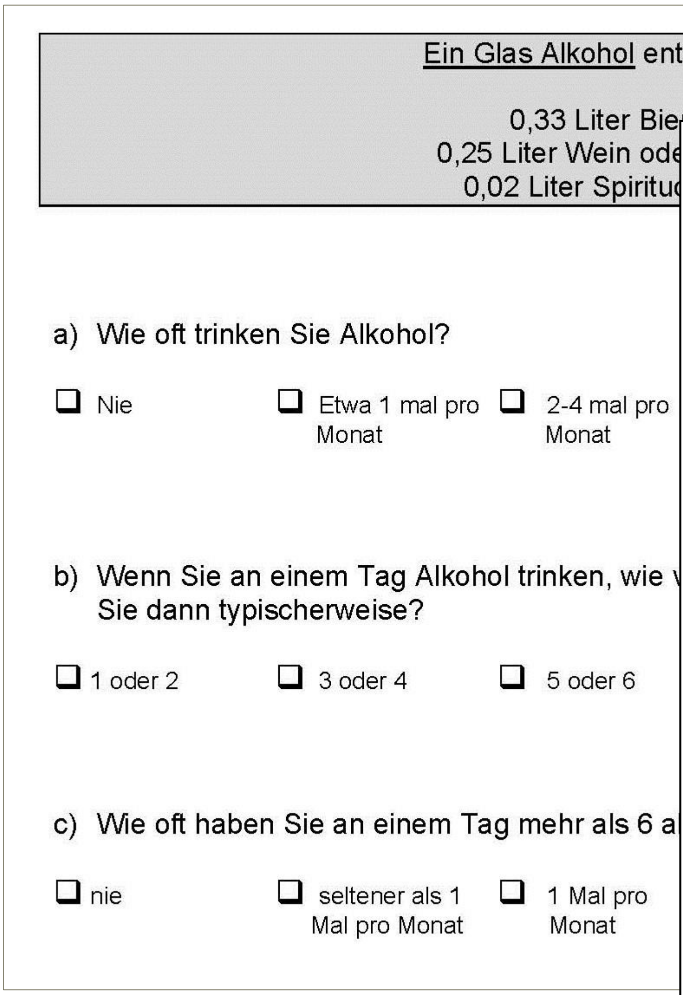
Ein Glas Alkohol entspricht:

0,33 Liter Bier
0,25 Liter Wein ode
0,02 Liter Spiritu

a) Wie oft trinken Sie Alkohol?

❑ Nie ❑ Etwa 1 mal pro Monat ❑ 2-4 mal pro Monat

b) Wenn Sie an einem Tag Alkohol trinken, wie v
Sie dann typischerweise?

❑ 1 oder 2 ❑ 3 oder 4 ❑ 5 oder 6

c) Wie oft haben Sie an einem Tag mehr als 6 a

❑ nie ❑ seltener als 1 Mal pro Monat ❑ 1 Mal pro Monat

A glass of pure alcohol corresponds to:
0.33 liter of beer
0.25 liter wine or sparkling wine
0.02 liter spirits

How often do you have a drink containing alcohol?

Never
Monthly or less
2-4 times a month
2-3 times a week
4 or more times a week

How many standard drinks containing alcohol do you have on a typical day?

1-2
3-4
5-6
7-9
10 or more

How often do you have six or more drinks on one occasion?

Never
Less than monthly
Monthly
Weekly
Daily or almost daily

Please turn the page!

Fig. 2: Extract from the information about brief intervention published by the Bundeszentrale für gesundheitliche Aufklärung (BzgA):

Intervention bei riskantem Konsum

Untersuchungsbefunde mitteilen:
Informieren Sie Ihren Patienten bzw. Ihre Patientin über die Untersuchungsbefunde. Vermeiden Sie dabei Pathologisierungen und Übertreibungen.

Trinkmenge festlegen:
Empfehlen Sie die Verringerung der Trinkmenge. Drängen Sie dabei nicht, sondern versuchen Sie partnerschaftlich Ziele für die Reduzierung festzulegen.

Ziele entwickeln:
Legen Sie zusammen (möglichst schriftlich) Menge und Frequenz des angstrebten Alkoholkonsums fest sowie die Schritte zur Zielerreichung in zeitlichen Stufen.

Empfe
zum A
- 2 al
- keir
- Alkc Schl
- Pau (z.B
- nur Dur
- nur
- nich
- keir
- keir
- keir

Sozial
Erarbe
Ihrer P
persör
geben

Zieler
Prüfer
der Ko
Sie die
Heben
Sie Lob

Bleibe

Weiter
Beratu
Verwe
kosten
über: v
„Alles
„Alkoh

Intervention for patients with hazardous alcohol consumption.

Communication of examination results:
Inform your patient about the examination results.
Avoid pathologizations and exaggerations.

Determine amount of drinking:
Recommend a reduction in the amount of drinking.
Do not push but try to arrange goals in partnership.

Arrange goals:
Define together the amount and frequency of the intended alcohol consumption (if possible, in writing) and determine intermediate stages with fixed deadlines.

Recommendations to reduce alcohol consumption:
Two alcoholic beverages per week maximum.
No stockpiling of alcohol.
Alcohol drinking only slowly and in small sips.
Take an interruption between two drinks (for instance one hour)
Use only non-alcoholic beverages for quenching thirst.
Drink alcohol only in social settings.
Not more than 1-3 glasses.
No alcohol on an empty stomach.
No alcohol before 5pm.
No alcohol after midnight.

Activating social settings:
Clarify with your patient who of his or her personal environment would be ready to support the patient.

Checking the achievement of drinking goals:
Check the compliance regarding drinking goals together and modify them possibly.
Name successes and praise the patient for them.
Keep in contact.

Information about additional knowledge and advice:
In your consultation refer to cost-free advice booklets (ordering via: www.bzga.de ...).

Please turn the page!

Please imagine the following situation:

One of your patients comes to your general practice. The consultation raised the suspicion of excessive alcohol consumption

Now we would like to know to what extent you would be ready to treat this patient with hazardous alcohol consumption with brief intervention including information about the treatment (about 20 minutes).

Please imagine you would receive for the brief intervention a (fictitious) reimbursement of 18 euros (Condition 1: 18€, Condition 2: 36€).

Please indicate to what extent you agree with the following statements.	do not agree					do agree
1) For the depicted financial reimbursement, I would inform any patient with excessive alcohol consumption about the intervention.	1	2	3	4	5	6
2) For the depicted financial reimbursement, I would carry out the intervention for any patient agreeing to participate.	1	2	3	4	5	6
3) The intervention is not feasible in my own general practice.	1	2	3	4	5	6
4) The intervention is not effective to reduce the alcohol consumption of excessive drinkers.	1	2	3	4	5	6

Next we want to know how capable you feel treating alcohol problems in your general practice.

Please indicate to what extent you agree with the following statements.

	do not agree					do agree
1. I feel I know enough about the causes of drinking problems to carry out my role when working with drinkers.	1	2	3	4	5	6
2. I feel I can appropriately advise my patients about drinking and its effects.	1	2	3	4	5	6
3. I feel I do not have much to be proud of when working with drinkers.	1	2	3	4	5	6
4. All in all I am inclined to feel I am failure with drinkers.	1	2	3	4	5	6
5. Pessimism is the most realistic attitude to take toward drinkers.	1	2	3	4	5	6
6. I feel I have the right to ask patients questions about their drinking when necessary.	1	2	3	4	5	6
7. I feel that my patients believe I have the right to ask them questions about drinking when necessary.	1	2	3	4	5	6
8. In general, it is rewarding to work with drinkers.	1	2	3	4	5	6
9. In general, I like drinkers.	1	2	3	4	5	6
10. I want to work with drinkers.	1	2	3	4	5	6

At last we would like to ask you for the following demographic data:
.

Your age in years: ______

Are you female ◯ or male ◯ ?

Do you work in a private practice ◯ , in a group practice ◯ ,

or in a medical care center ◯ ?

The environment of your practice is rather urban ◯ or rather rural ◯.

Your practice has been established since: ______

You have around ______ treatments in the quarter.

The proportion of your patients with an acute alcohol problem is around ____ %.

Thanks for your support!
