# Supplementary material for: Financial reimbursement - irrelevant for GPs’ readiness to implement brief intervention to reduce alcohol consumption? A cross-sectional vignette study
Source: BMC Fam Pract. 2020 Aug 19;21:170. doi: 10.1186/s12875-020-01231-9 (PMC7439686; doi:10.1186/s12875-020-01231-9)
Supplement: Supplementary file 2 — Additional file 2. [file 12875_2020_1231_MOESM2_ESM.pdf]

Gefördert durch:

Bundesministerium für Gesundheit

aufgrund eines Beschlusses des Deutschen Bundestages

Martin-Luther-Universität Halle-Wittenberg

FACHHOCHSCHULE FÜR GESUNDHEIT
SRH GERA

Fb-Nr - _ _ _ -

Dear Colleague,

We would like to thank you for supporting us in our research.

For years, the World Health Organization (WHO) and other institutions have called on General Practitioners to get more involved in the treatment of patients with problematic alcohol consumption. The goal of our research is a critical review of presently available treatment options for those patients with special attention to feasibility in routine care. We would like to know what you think about the presently available treatment options designed for general practice.

Filling out our questionnaire takes around 10 minutes.
Please complete our questionnaire from front to back.

We are at your disposal for any questions.

Prof. Dr. phil. Thomas Fankhänel [1,2], Prof. Dr. med. Andreas Klement [1]

[1] Martin-Luther-Universität Halle-Wittenberg
Medizinische Fakultät, Sektion Allgemeinmedizin
Magdeburger Str. 8, D-06112 Halle

[2] Studiengang Gesundheitspsychologie
SRH Fachhochschule für Gesundheit Gera
Neue Straße 28-30, 07548 Gera
Telefon: +49 365 773407-41
Telefax: +49 365 773407-77

For years, the WHO and other institutions have called on General Practitioners (GPs)_ to get more involved in the treatment of patients with excessive alcohol consumption.

GPs should systematically screen all of their patients for their alcohol consumption levels. If an excessive consumption level is suggested (termed hazardous alcohol consumption) the patient should be informed about suitable treatment options.

For instance, patients with the intention of reducing their alcohol consumption level can be informed about a pharmacological treatment. Here they should learn that such a drug may influence brain processes so that cravings for alcohol will be reduced.

On the next page you will find an example of a press release informing about the market launch of such a drug.

Please take a close look at the information.

Please turn the page!

Fig.: Extract from a press release about the market launch of a new drug for the reduction of alcohol consumption.

Kein revolutionär neuer Wirkstoff

„Bei Nalmefen handelt es sich nicht um einen revolutionär neuen Wirkstoff, sondern um ein 20 Jahre altes Konzept, das auf der Blockade von Opiatrezeptoren beruht“, sagt **Prof. Dr. Rainer Spanagel**, wissenschaftlicher Direktor am Zentralinstitut für Seelische Gesundheit in Mannheim, das an den 3 placebokontrollierten Phase-3-Studien für die Zulassung beteiligt war [1,2,3]. Nalmefen unterscheide sich nicht grundlegend von Naltrexon, das zur Rückfallprophylaxe von Alkoholkranken eingesetzt wird, erläutert der Pharmakologe Spanagel gegenüber *Medscape Deutschland*.

Absolut neu sei jedoch, dass das Medikament in einer Art Notfall nach Bedarf zur Reduktion des Alkoholkonsums – und nicht zur Erhaltung der Totalabstinenz eingesetzt wird, sagt Spanagel. So nimmt [illegible] er das Bedürfnis verspürt, Alkohol zu trinken – 1 bis 2 Stunden vor dem [illegible] ansonsten möglichst nach dem Beginn.

„Dem Patienten wird dann nicht mehr bei jedem Rückfall suggeriert, da[illegible] dafür gelobt, dass er weniger trinkt“, betonte Spanagel: „Das ist ein Par[illegible] Alkoholabhängigkeit.“ Da mit dem übermäßigen Alkoholkonsum über 2[illegible] das Risiko zu erkranken, linear mit der Trinkmenge steige, wäre es sch[illegible] Patienten nur noch die Hälfte der bisherigen Alkoholmenge trinken, so [illegible]

Die Zielgruppe sind laut Spanagel nicht die schweren Alkoholiker, die [illegible] hinter sich gebracht haben, sondern die Gruppe der Personen mit risk[illegible] Alkoholkonsum – mindestens 1 Million Menschen in Deutschland.

Not a revolutionary new ingredient.

"Nalmefene is not a revolutionary new ingredient, but a 20 year old concept based on the blocking of opiate receptors", says **Prof. Dr. Rainer Spanagel**, Scientific Director of the Zentralinstitut für seelische Gesundheit in Mannheim that was involved in the three placebo-controlled Phase 3 studies for the approval. Spanagel told Medscape Deutschland that Nalmefene is not fundamentally different from Naltrexone, which is already being used for the prevention of relapse in alcoholics.

Spanagel says however it would be completely new that a drug can be taken in emergencies and as needed in order to reduce alcohol consumption, but not to maintain total abstinence. The pill should be taken only when the patient feels a need for alcohol - 1 or 2 hours before the expected intake, otherwise after the beginning.

"It will no longer be suggested that the patients have failed any time they relapse, instead they will be praised because of drinking less", says Spanagel: That is a paradigm shift in the treatment of alcohol dependency.

Excessive alcohol consumption is associated with around 200 comorbidities and the risk of getting sick increases with the amount of drinking linearly. It would be thus a great success if those patients would drink only the half of their previous amount says Spanagel.

According to Spanagel, the target group wouldn't be heavy alcoholics who may have had several unsuccessful withdrawals behind them, but the group of persons with hazardous and harmful alcohol consumption - at least 1 Million people in Germany.

Please turn the page!

Please imagine the following situation:

One of your patients comes to your general practice. The consultation raised the suspicion of excessive alcohol consumption

Now we would like to know to what extent you would be ready to treat this patient with hazardous alcohol consumption with pharmacological intervention including information about the treatment (about 20 minutes).

Please imagine you would receive for the brief intervention a (fictitious) reimbursement of 18 euros (Condition 1: 18€, Condition 2: 36€).

Please indicate to what extent you agree with the following statements.	do not agree					do agree
1) For the depicted financial reimbursement, I would inform any patient with excessive alcohol consumption about the intervention.	1	2	3	4	5	6
2) For the depicted financial reimbursement, I would carry out the intervention for any patient agreeing to participate.	1	2	3	4	5	6
3) The intervention is not feasible in my own general practice.	1	2	3	4	5	6
4) The intervention is not effective to reduce the alcohol consumption of excessive drinkers.	1	2	3	4	5	6

Next we want to know how capable you feel treating alcohol problems in your general practice.

Please indicate to what extent you agree with the following statements.

	do not agree					do agree
1. I feel I know enough about the causes of drinking problems to carry out my role when working with drinkers.	1	2	3	4	5	6
2. I feel I can appropriately advise my patients about drinking and its effects.	1	2	3	4	5	6
3. I feel I do not have much to be proud of when working with drinkers.	1	2	3	4	5	6
4. All in all I am inclined to feel I am failure with drinkers.	1	2	3	4	5	6
5. Pessimism is the most realistic attitude to take toward drinkers.	1	2	3	4	5	6
6. I feel I have the right to ask patients questions about their drinking when necessary.	1	2	3	4	5	6
7. I feel that my patients believe I have the right to ask them questions about drinking when necessary.	1	2	3	4	5	6
8. In general, it is rewarding to work with drinkers.	1	2	3	4	5	6
9. In general, I like drinkers.	1	2	3	4	5	6
10. I want to work with drinkers.	1	2	3	4	5	6

At last we would like to ask you for the following demographic data:
.

Your age in years: ______

Are you female ◯ or male ◯ ?

Do you work in a private practice ◯ , in a group practice ◯ ,

or in a medical care center ◯ ?

The environment of your practice is rather urban ◯ or rather rural ◯.

Your practice has been established since: ______

You have around ______ treatments in the quarter.

The proportion of your patients with an acute alcohol problem is around ____ %.

Thanks for your support!
